# Imbalances in the Content of Sleep and Pain Assessments in Patients with Chronic Pain: A Scoping Review

**DOI:** 10.12688/f1000research.166110.3

**Published:** 2025-12-12

**Authors:** Katsuyoshi Tanaka, Yuichi Isaji, Kosuke Suzuki, Kohei Okuyama, Yasuyuki Kurasawa, Masateru Hayashi, Takashi Kitagawa

**Affiliations:** 1 Department of Physical Therapy, School of Health Sciences, Bukkyo University, Kyoto, Kyoto, 604-8418, Japan; 2 Department of Rehabilitation, Yamagata Saisei Hospital, Yamagata, Yamagata, Japan; 3 Department of Rehabilitation, Faculty of Health Science, Nagano University of Health and Medicine, Nagano, Nagano, Japan; 4 Department of Rehabilitation, Matsuoka Orthopedic Surgery and Internal Medicine Rehabilitation, Gifu, Gifu, Japan; 5 Department of Physical Therapy, School of Health Sciences, Shinshu University, Matsumoto, Nagano, Japan

**Keywords:** pain, sleep disturbances, assessment tools

## Abstract

**Background:**

Sleep disturbances frequently occur in concomitance with chronic pain, exacerbating its detrimental effects and diminishing patients’ quality of life. Although various studies have explored the relationship between chronic pain and sleep disturbances, comprehensive evidence on detailed assessment methods and their bidirectional interactions remains limited. This scoping review aimed to examine the characteristics and prevalence of assessment methods for sleep and pain-related outcomes in individuals with chronic pain.

**Methods:**

A comprehensive search of nine databases identified observational and interventional studies examining the relationship between sleep disturbances/disorders and chronic pain in adults. A literature search was conducted in MEDLINE, the Cochrane Central Register of Controlled Trials, Embase, PsycINFO, Web of Science, Cumulative Index to Nursing and Allied Health Literature (CINAHL) as well as gray literature sources, Open Grey. In addition, the following trial registries were searched for ongoing or unpublished trials: the World Health Organization International Clinical Trials Registry Platform and ClinicalTrials.gov.

**Results:**

This review included 81 of 3,513 studies. Approximately 90.1% of studies relied on self-report sleep assessments, whereas only 9.9% incorporated objective measures. Additionally, 7.4% of studies used a combination of self-report and objective sleep assessments. The visual analog and numeric rating scales were the most frequently used methods for assessing pain-related outcomes (59.3%). Despite extensive research on sleep and chronic pain, critical gaps persist, particularly in the integration of multidimensional assessment tools.

**Conclusions:**

This scoping review discovered imbalances in the content of both sleep and pain assessments. Future studies should integrate both objective and self-report assessment tools to provide a more comprehensive understanding of this interaction.

**Abbreviations**
BPI: Brief Pain Inventory
ISI: Insomnia Severity Index
NRS: Numeric Rating Scale
PSQI: Pittsburgh Sleep Quality Index
PRO: Patient-Reported Outcome
QOL: Quality Of Life
RCT: Randomized Controlled Trial
ScR: Scoping Review
VAS: Visual Analog Scale

## 1. Introduction

Pain is a fundamental human experience that functions as a protective mechanism, however, when it persists beyond the expected period of tissue healing, it is regarded as a pathological condition known as chronic pain. The World Health Organization recognizes chronic pain as a disease, making it one of the most prevalent conditions worldwide. ^1^ Chronic pain results in significant disability and imposes a substantial economic strain on society. ^2^ In addition to persistent pain, individuals with chronic pain experience various consequences, including deterioration in the quality of life (QOL), higher prevalence of depressive symptoms, and greater levels of disability compared with those without pain. ^3^ The financial impact of chronic pain, including healthcare expenses and reduced work efficiency, is substantial. ^4^ This underlines the extensive influence of pain on the individual and the community as a whole. Furthermore, chronic pain often coexists with sleep disturbances, which exacerbate the adverse effects of pain, adding to the overall strain on individuals and society. ^5, 6^

Patients with chronic pain frequently develop sleep disturbances. ^7– 10^ Sleep disturbance including inadequate sleep, insomnia and obstructive sleep apnea, represent significant and widespread health concerns. ^11, 12^ Notably, the prevalence of sleep disturbance is high among patients with chronic musculoskeletal pain, affecting approximately 75% and 44% of such individuals, respectively. ^9, 13^ Previous studies have suggested a correlation between compromised sleep and reduced QOL, adverse general health outcomes, elevated levels of depression, and diminished physical function. ^14^ Additionally, the concomitance of chronic pain and sleep disturbances leads to further deterioration in overall health and QOL. A bidirectional association has also been suggested, wherein pain negatively affects sleep, and sleep disturbances contribute to increased pain. ^15^

Polysomnography, which is considered the gold standard for the objective assessment of sleep, has been used in various chronic pain conditions, such as fibromyalgia, rheumatoid arthritis, osteoarthritis, and temporomandibular pain. ^7, 16^ In addition to polysomnography, sleep assessment, encompassing sleep duration and quality, has been conducted using several tools, such as actigraphy, questionnaires, and wearable devices. ^17, 18^ Moreover, although several studies have investigated the relationship between sleep and chronic pain, most existing reviews have focused on specific populations, such as those with postsurgical pain, pediatric pain, or low back pain. ^19– 21^ Thus, comprehensive evidence on detailed methods for assessing the relationship between chronic pain and sleep disturbance remains scarce.

Therefore, this scoping review (ScR) aimed to examine the characteristics and prevalence of methods used to assess sleep and pain-related outcomes in individuals with chronic pain and to identify gaps in the evidence, with the objective of guiding future studies.

## 2. Methods

This ScR was conducted according to the Joanna Briggs Institute methodology for scoping reviews, following all eight recommended steps without deviation. ^22^ The study was registered with the Open Science Framework ( https://doi.org/10.17605/OSF.IO/5JK63) on March 29, 2024. This review also adhered to the Preferred Reporting Items for Systematic Reviews and Meta-Analyses extension for Scoping reviews (PRISMA-ScR) checklist. The inclusion criteria were established based on the participants, concept, and context of the study.

### 2.1 Eligibility criteria

This ScR included studies on individuals with chronic pain lasting for >3 months. Studies on individuals with malignancy-related or cancer-related pain or acute pain conditions, such as postoperative pain, were excluded. Moreover, studies that included children (≤18 years) and/or participants with other conditions besides chronic pain were excluded. No restrictions were imposed with respect to the sex, location, race, country, or language of the participants. This review evaluated the measurement tools used for sleep assessment in individuals with chronic pain conditions, including polysomnography, wearable devices, and questionnaires. Additionally, we identified various types of pain-related assessments, including pain intensity, severity, disability, catastrophizing, threshold, and tolerance. In other words, we included studies that involved sleep assessments in individuals with chronic pain conditions.

This ScR included randomized controlled trials (RCTs), crossover trials, quasi-RCTs, non-RCTs, cross-sectional studies, and prospective and retrospective cohort studies, encompassing both observational and interventional designs. Protocols and conference abstracts were included in the initial screening, with a secondary screening conducted to verify the existence of published articles. Case reports, case-control studies, systematic reviews, meta-analyses, and narrative reviews were excluded.

### 2.2 Search strategy

The search strategy was designed to identify both published and unpublished studies. A literature search was conducted in MEDLINE, the Cochrane Central Register of Controlled Trials, Embase, PsycINFO, Web of Science, Cumulative Index to Nursing and Allied Health Literature (CINAHL) as well as gray literature sources, Open Grey. In addition, the following trial registries were searched for ongoing or unpublished trials: the World Health Organization International Clinical Trials Registry Platform and ClinicalTrials.gov.

The text words found in the titles and abstracts of relevant articles, along with the index terms used to describe the articles, were used to develop a comprehensive search strategy across nine databases (the complete PubMed search strategy is provided in Table S1). Previous studies were also referenced. ^19, 23, 24^ Studies published in any language were included, with no restrictions on the publication date. The final comprehensive search was conducted on March 29, 2024.

### 2.3 Study selection and source of evidence

All identified citations were collated and uploaded into Rayyan (Qatar Computing Research Institute, Ar Rayyan, Qatar, https://www.rayyan.ai/), and duplicates were removed. Following a pilot test, two or more independent reviewers (K.T. and Y.I.) screened the study titles and abstracts based on the eligibility criteria. The full text of relevant sources was retrieved, and their citation details were imported into Rayyan. Two or more independent reviewers (K.T., Y.I., K.S., M.H., K.O., and Y.K.) assessed the full text of the selected studies based on the eligibility criteria. The reasons for excluding sources that did not meet the eligibility criteria were documented and reported in this ScR. Any disagreements between reviewers at each stage of selection were resolved through discussion or by consulting additional reviewers.

The results of the search and study inclusion process were comprehensively reported in the final ScR and illustrated in a Preferred Reporting Items for Systematic Reviews and Meta-Analyses extension for ScR flow diagram. ^25^

### 2.4 Data extraction

Data were extracted from the included studies using Microsoft Excel (Microsoft Corp., Redmond, WA, USA) by the first author (K.T.), with the assistance of ChatGPT-4o (San Francisco, CA, USA) and NotebookLM (Mountain View, CA, USA). ^26, 27^ These AI tools were used to facilitate the extraction and preliminary organization of information from the included studies. All outputs generated by AI were reviewed and verified against the original sources by the first author (K.T.) to ensure accuracy, and all final decisions were made by the research team. No dedicated systematic review software (e.g., Covidence) was used for data extraction. The extracted data included the first author’s name, country of origin, study design, sample size, participant characteristics (age, sex, and diagnosis), and tools used for assessing sleep disturbance and pain-related outcomes. The draft data extraction tool was modified and refined as necessary throughout the data extraction process. Where necessary, the authors of the included studies were contacted to obtain any missing or additional data.

To enhance reproducibility, the exact data extraction template has been made publicly available in the Open Science Framework repository ( https://doi.org/10.17605/OSF.IO/5JK63). The column headings include, for example, “Title”, “Author”, “Year of publication”, “Country”, “Study design”, “Diagnosis/Conditions”, “Sample size”, “Age”, “Sleep assessment tool”, and “Assessment tools of pain-related factor”. Verification checks included double-checking the counts of sleep and pain assessment tools extracted by AI against both the full-text tables and textual descriptions, and cross-referencing the extracted sample characteristics with the methods sections of the original papers.

### 2.5 Sensitivity statement on search recency

The literature search was last conducted on March 29, 2024. To contextualize the recency of the review, an updated search was performed in PubMed using the same search strategy, limited to publications from 2024 to 2025, on November 7, 2025. This additional search identified 131 new records. Although this increase indicates an ongoing rise in research activity, particularly in wearable-based sleep assessments, the number of new studies remains modest relative to the total volume of literature included in the present review. Therefore, the findings of this review are considered broadly representative of the current evidence base, while future updates should incorporate these recent studies to capture emerging trends.

## 3. Results

### 3.1 Study selection

**Figure 1.**
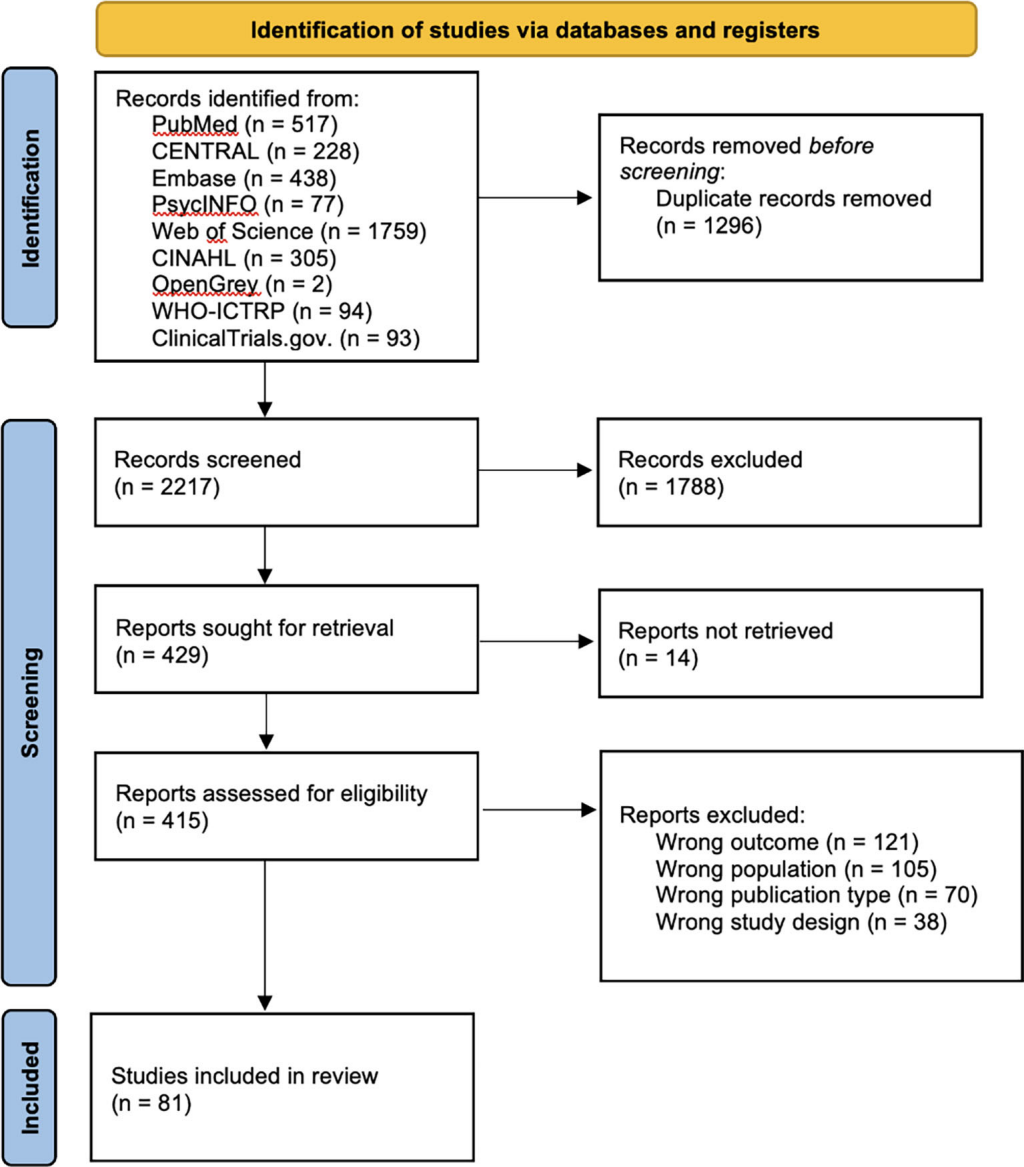
PRISMA flow diagram illustrating the structure of the search and screening process.

A total of 3,513 articles were retrieved during the database search. After eliminating 1,296 duplicates, the titles and abstracts of 2,217 articles were screened. Thereafter, the full texts of the remaining 415 articles were assessed for eligibility. Ultimately, only 81 studies that met the eligibility criteria were included in the analysis ( Figure 1).

### 3.2 Study and participant characteristics

Of the 81 included studies, 26 (32.1%) were RCTs, ^28– 53^ while the remaining studies were non-RCTs. ^54– 108^ The included studies were published between 1998 and 2024, with nearly half (45.7%, 37 of 81) published within the last 5 years. ^30– 32, 34– 36, 40, 41, 43, 48, 51– 54, 58, 59, 63, 67, 68, 73, 76, 78– 82, 85– 87, 90– 93, 95, 96, 101, 106^ The distribution of participants varied across studies: 31 studies (38.3%) focused on examining patients with chronic pain, irrespective of pain type, accounting for 40.5% of the total participants (7,413 out of 18,316). ^32, 38, 42, 47, 51, 52, 54, 57, 58, 65, 67, 69– 73, 76, 79, 80, 82, 85, 88, 89, 91, 92, 94, 97, 99, 101, 103, 106^ Eleven studies (13.6%) investigated patients with chronic low back pain, accounting for 12.4% of the total participants (2,266 participants). ^33, 36, 46, 48, 56, 81, 90, 95, 98, 107, 108^ Eight studies (9.9%) examined patients with chronic musculoskeletal pain, irrespective of location, accounting for 9.1% of the total participants (1,667 individuals). ^41, 62– 64, 77, 78, 86, 105^ Six studies (7.4%) assessed patients with chronic neck pain, comprising 5.2% of the total participants (950 participants). ^34, 40, 60, 61, 96, 100^

To provide an overview of demographic and clinical characteristics of the study populations, Table 1 summarizes the participant characteristics across the included studies, while detailed study-level information is available in Supplementary Table S2.

**Table 1.** Summary of included studies and participant characteristics.

Category	N (%) or Median (IQR)
Study design	
RCTs	26 (32.1%)
non-RCTs	55 (67.9%)
Publication period	1998–2024 (45.7% after 2019)
Age (years)	48.1 (44.0-54.1) *
Female participants (%)	61.4 (53.4-72.5) **
Pain type	
Chronic pain	31 (38.3%)
CLBP	11 (13.6%)
Chronic musculoskeletal pain	8 (9.9%)
CNP	6 (7.4%)
Chronic pain and insomnia	3 (3.7%)
CTTH	2 (2.5%)
PHN	2 (2.5%)
Fibromyalgia or CLBP	1 (1.2%)
Idiopathic chronic pain	1 (1.2%)
Chronic spinal degenerative disease	1 (1.2%)
Traumatic SCI	1 (1.2%)
ICONP and MMP	1 (1.2%)
Chronic Migraine	1 (1.2%)
CLBP, and unspecified back pain	1 (1.2%)
Orofacial pain	1 (1.2%)
Chronic back pain	1 (1.2%)
Insomnia, chronic musculoskeletal pain	1 (1.2%)
TMD	1 (1.2%)
Chronic pain with insomnia	1 (1.2%)
Chronic pain after SCI	1 (1.2%)
Osteoarthritis of the knee	1 (1.2%)
CNP, CLBP, and/or generalized pain	1 (1.2%)
NSCNP	1 (1.2%)
NSCSP and comorbid insomnia	1 (1.2%)
Masticatory/cervical muscle pain or temporomandibular joint pain	1 (1.2%)

^*^ Reported in 79 studies.

^**^ Reported in 74 studies.

### 3.3 Outcome measurements

An overview of the included studies is presented in supplemental materials (Table S2, S3). Various methods have been used to assess sleep disturbances and pain-related outcomes, with patient-reported outcomes (PROs) being the most frequently utilized.

**Figure 2.**
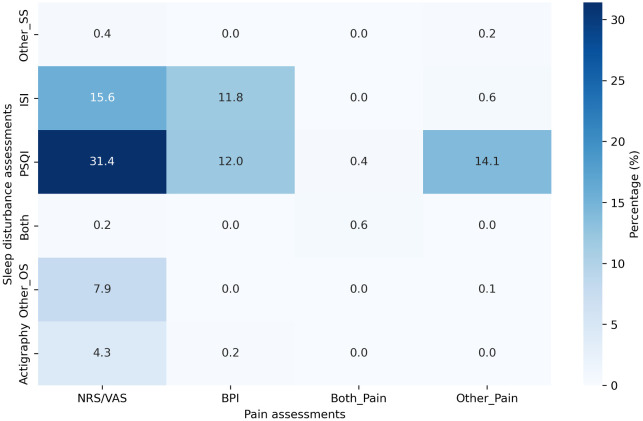
Percentage of the combined use of sleep problem and pain-related outcome assessments.

The Pittsburgh Sleep Quality Index (PSQI) (45 of 81 studies, 55.6%) and Insomnia Severity Index (ISI) (21 of 81 studies, 25.9%) were the most commonly used tools for the assessment of sleep problems ( Figure 2). Other self-reported sleep assessment methods used included the Athens Insomnia Scale and sleep diaries. Sleep problems were predominantly assessed based on the participant’s entries in sleep diaries. In terms of study methodology, 73 of 81 studies (90.1%) relied solely on PROs (90.1% [73/81]). ^30– 53, 60– 108^ Only 2 of 81 studies (2.5%) relied solely objective assessments, ^58, 59^ such as actigraphy, whereas 6 of 81 studies (7.4%) used a combination of PROs and objective assessments. ^28, 29, 54– 57^ Other objective sleep assessment methods used included polysomnography and electroencephalography.

For PRO assessments, the outcomes were frequently evaluated using the numeric rating scale (NRS) or visual analog scale (VAS) (59.3% [48/81]). ^32, 34– 41, 44, 45, 49, 50, 52, 53, 55– 62, 64– 66, 70– 72, 77, 79, 81, 85, 87, 88, 90, 91, 94– 98, 100, 103, 105, 107, 108^ The Brief Pain Inventory (BPI) was the second most frequently used pain assessment tool (28.4% [23/81]). ^28, 30, 31, 33, 42– 44, 46, 47, 51, 57, 63, 67– 69, 73, 76, 78, 80, 82, 86, 89, 99^ Additional pain-related outcome measures included the Multidimensional Pain Inventory and Pain Disability Questionnaire. Psychometric factors were evaluated in more than half of the included studies (60.5% [49/81]). ^30, 32, 35– 38, 41, 42, 44, 47, 49, 50, 52, 54, 55, 58, 60– 63, 69– 79, 83– 85, 87, 90– 95, 97– 99, 102, 103, 105, 106, 108^ Objective pain assessment was rarely performed, with only one study utilizing quantitative sensory testing. ^98^

Figure 2 presents the detailed percentages of the combined use of sleep problems and pain-related outcome assessments. The NRS/VAS was frequently used in combination with the PSQI (31.4%), followed by combination of the NRS/VAS with the ISI (15.6%). The simultaneous use of both self-report and objective sleep assessments, along with the NRS or VAS and the BPI, was extremely rare, occurring in only 0.6% of studies ( Figure 2, Table 2).

**Table 2.** Mapping of pain domains to assessment tools idendified in the included studies.

Pain domains	Assessment tools	Frequency, n (%)
Pain Intensity	Total studies assessing this domains	71 (87.7%)
	Numeric Rating Scale (NRS)	33 (40.8%)
	Visual Analog Scale (VAS)	15 (18.5%)
	Brief Pain Inventory (BPI) *	23 (28.4%)
Interference/Disability	Total Studies assessing this domain	46 (56.8%)
	Brief Pain Inventory (BPI) *	23 (28.4%)
	Pain Disability Questionnaire (PDQ)	10 (12.3%)
	Multidimensional Pain Inventory (MPI)	7 (8.6%)
	Region-specific (ODI, RMDQ, NDI, etc.)	6 (7.4%)
Pain Quality	McGill Pain Questionnaire (MPQ, SF-MPQ)	5 (6.2%)
Psychosocial Factors	Total Studies assessing this domain	49 (60.5%)
	Depression/Anxiety Scales (HADS, BDI, PHQ, PASS, etc.)	44 (54.3%)
	Pain Catastrophizing Scale (PCS)	18 (19.8)
	Tampa Scale for Kinesiophobia (TSK)	6 (6.6%)
	Self-Efficacy/Acceptance (PSEQ, CPAQ)	2 (2.5%)
Quantitative Sensory Testing (QST)	Pressure Pain Threshold (PPT)	1 (1.2%)

^*^ BPI was mapped to both “Pain Intensity” and “Pain Interference” domains as it assesses both pain severity and its impact on daily functions.

### 3.4 Association between sleep and pain outcomes

Although a meta-analysis was not conducted, a narrative summary of the included studies reveals distinct patterns in the relationship between sleep and pain.

Studies relying on patient-reported outcomes consistently demonstrated a significant positive correlation, where higher score on sleep questionnaires (e.g., PSQI, ISI) were associated with greater pain intensity or interference.

In contrast, findings from objective assessments varied depending on the measurement modality. Studies utilizing movement-based devises, such as actigraphy, generally did not show a clear association with pain intensity or other pain-related outcomes. However, objective measures based on physiological parameters, such as heart rate or electroencephalography (EEG), tended to exhibit significant associations with pain outcomes.

## 4. Discussion

This ScR highlighted the use of various measurement tools for assessing sleep and pain. The review emphasized the diversity of assessment tools used to evaluate sleep and pain, revealing substantial inconsistencies and the lack of standardization. Despite the large body of research on this topic, critical gaps persist, particularly the absence of generalizable objective measurements for sleep assessment, which may hinder the reliability and applicability of the current findings.

A key finding of this review is the predominant reliance on self-report measures for evaluating sleep disturbances. Over 90% of the included studies utilized PROs, such as the PSQI or the ISI. Although these tools provide practical and accessible methods of assessing perceived sleep quality, they are inherently limited by individual biases and self-reported variability. ^109, 110^ In contrast, objective measures such as actigraphy and polysomnography provide precise and quantifiable data on sleep architecture, including sleep stages, latency, and fragmentation. ^111, 112^ However, these tools were employed by only a small portion of the included studies, with objective methods utilized by 9.9% (8/81) of studies. This imbalance likely stems from the challenges, which traditionally include cost and other constraints, hindering the use of objective measures, thereby complicating the comprehensive assessment of sleep disturbances. ^113, 114^ Furthermore, our review identified a discrepancy in the results derived from different objective modalities. While movement-based assessment (e.g., actigraphy) often failed to correlate with pain, physiological measures (e.g., EEG, heart rate) consistently showed significant associations. This suggests that while pain may not always disrupt gross body movements during sleep, it likely impacts physiological sleep architecture and autonomic regulation. Future research should prioritize the integration of these objective tools to provide a more robust understanding of sleep disturbances in individuals with chronic pain.

Beyond sleep assessment, this review also identified variability and potential bias in the pain-related outcome evaluations. Most studies focused primarily on examining pain intensity, often measured using the NRS or the VAS. Although these tools are widely used and validated, ^115, 116^ they only capture one aspect of the complex pain experience. ^117– 119^ Approximately half of the studies assessed the psychological factors associated with pain, but other important domains, such as pain-related disability and sensitization, were less frequently explored. Notably, only one study included in this review utilized quantitative sensory testing. ^98^ This limited focus restricts the understanding of pain mechanisms and hinders the development of targeted treatment strategies. More comprehensive pain assessment protocols that incorporate these additional dimensions are necessary to produce clinically relevant evidence. ^120^

The complex relationship between sleep disturbances and chronic pain necessitates a multidimensional research approach. Current evidence emphasizes a strong bidirectional relationship between sleep and pain, with sleep disturbances often predicting future pain severity more robustly than pain predicts sleep problems. ^6^ Experimental studies have further demonstrated that sleep deprivation directly exacerbates pain perception, lowering pain thresholds and increasing hyperalgesia. ^121^^,^^122^ These interactions are hypothesized to be mediated by shared neurobiological mechanisms, particularly central sensitization and impairments in descending pain inhibitory pathways, which nociceptive signaling. ^123, 124^ Incorporating objective sleep assessments and multidimensional pain measures into research and practice will enhance the quality of evidence and support the development of precision medicine approaches tailored to individual patient needs. Furthermore, interdisciplinary collaboration that brings together experts in neurology, psychology, and bioinformatics could facilitate the development of innovative assessment tools and therapeutic interventions.

This review has some limitations. The lack of synthesis of the findings restricted our ability to evaluate methodological rigor and the overall reliability of the evidence. Additionally, the possibility of overlooking relevant studies cannot be completely excluded, which may have introduced selection bias. However, this review included gray literature and non-English studies to mitigate this potential bias. A further limitation is that the literature search was last conducted in March 2024, and more recent studies may not have been captured.

## 5. Conclusions

This ScR highlights the imbalance in the characteristics of sleep and pain assessments, indicating the need for a more comprehensive evaluation of sleep disturbances and pain-related outcomes. Addressing the gaps in objective and multidimensional assessments could facilitate the development of personalized interventions that improve patient outcomes and overall quality of care.

## Ethics and consent

This scoping review did not involve human participants directly, and therefore ethical approval was not required.

## Data Availability

All data underlying the results are available in the Open Science Framework repository: ( https://doi.org/10.17605/OSF.IO/5JK63), licensed under CC0 1.0 Universal. ^125^ This includes the PRISMA-ScR checklist, flowchart, data for figure and supplementary tables.
