# Supplementary material for: Imbalances in the Content of Sleep and Pain Assessments in Patients with Chronic Pain: A Scoping Review
Source: F1000Res. 2025 Dec 12;14:605. Originally published 2025 Jun 20. [Version 3] doi: 10.12688/f1000research.166110.3 (PMC12759280; doi:10.12688/f1000research.166110.3)
Supplement: Peer review history

# Reviewer report: Reviewer response for version 3

**DOI:** 10.5256/f1000research.192814.r440945

**Published:** 2026-01-02

**Authors:** Yuri Chaves Martins, Peyton Murin

**Affiliations:** 1 Anesthesiology, Saint Louis University School of Medicine (Ringgold ID: 12274), St. Louis, Missouri, USA; 2 Neurology, Saint Louis University School of Medicine (Ringgold ID: 12274), St. Louis, Missouri, USA

**Response to Yuri Chaves Martins and Peyton J. Murin**

 We greatly appreciate the reviewer’s insightful comments. Our responses to your comments are given below.

**Introduction**

*Comment:* The transition between pain to chronic pain could be smoother. Would recommend rewording to allow for better flow/cohesion.

*Responses:*

 We appreciate the reviewer’s helpful suggestion. To improve the logical flow from pain to chronic pain, we have revised the beginning of the Introduction. Specifically, we added a sentence to clarify the conceptual transition from pain as a protective mechanism to chronic pain as a pathological condition. The following text was added:

“Pain is a fundamental human experience that functions as a protective mechanism, however, when it persists beyond the expected period of tissue healing, it is regarded as a pathological condition known as chronic pain.”

**2 ^nd^ review: The change in wording improved the flow of the introduction.**

**Methods**

*Comment:* You transparently describe using ChatGPT-4o and NotebookLM with human verification. To enhance reproducibility, please (i) provide the exact extraction template (e.g., the Excel column headings) in the OSF repository and (ii) add one or two concrete examples of verification checks you performed (e.g., double-checking instrument counts against full texts).

 The search was last run on March 29, 2024. You acknowledge that newer studies may be missing (page 7). Given the rapid growth in wearable-based sleep assessments, a short sensitivity statement (e.g., how many additional records a quick 2024–2025 update might add based on pilot scoping) would contextualize recency without requiring a full rerun.

*Responses:*

 We sincerely thank the reviewer for these valuable and constructive comments.

 (i) To improve transparence and reproducibility, we have updated the Excel data extraction template (showing the column headings used for data collection) to our Open Science Framework (OSF) repository ( https://doi.org/10.17605/OSF.IO/5JK63). The revised Methods section has showed the major data fields extracted (eg., author country, study design, and tools used for sleep and pain assessment). The following sentence was added:

“To enhance reproducibility, the exact data extraction template has been made publicly available in the Open Science Framework repository ( https://doi.org/10.17605/OSF.IO/5JK63 ). The column headings include, for example, “Title”, “Author”, “Year of publication”, “Country”, “Study design”, “Diagnosis/Conditions”, “Sample size”, “Age”, “Sleep assessment tool”, and “Assessment tools of pain-related factor”.”

 (ii) We have also added specific examples of verification procedures used during data validation. These included double-checking the counts of sleep and pain assessment tools extracted by AI against both the full-text tables and textual descriptions, and cross-referencing the extracted sample characteristics with the methods section. The following sentence was added:

“Verification checks included double-checking the counts of sleep and pain assessment tools extracted by AI against both the full-text tables and textual descriptions, and cross-referencing the extracted sample characteristics with the methods sections of the original papers.”

 (iii) To contextualize the recency of our research, we performed an updated PubMed search using the same search strategy as in the original review, limited to publications from 2024 to 2025, on November 7, 2025. This updated search identified 131 new records, reflecting a moderate increase in studies related to wearable-based sleep assessments. We have added a corresponding sensitivity statement to the Methods section, clarifying that the findings of the present review remain broadly representative of the current evidence base, while future updates should incorporate these recent studies. The following sentences were added:

“The literature search was last conducted on March 29, 2024. To contextualize the recency of the review, an updated search was performed in PubMed using the same search strategy, limited to publications from 2024 to 2025, on November 7, 2025. This additional search identified 131 new records. Although this increase indicates an ongoing rise in research activity, particularly in wearable-based sleep assessments, the number of new studies remains modest relative to the total volume of literature included in the present review. Therefore, the findings of this review are considered broadly representative of the current evidence base, while future updates should incorporate these recent studies to capture emerging trends.”

**2 ^nd^ Review: The above changes increased reproducibility of the methodology. These changes sufficiently addressed our concerns.**

**Results**

*Comment:* 3.2: Would recommend including more robust analysis on the gender distribution, age, pain type. Would recommend adding a table to summarize this data.

*Response*:

 Thank you for this helpful suggestion. In response, we strengthened the description of participant characteristics by providing an aggregated summary of age distribution, sex distribution, and pain diagnoses in the main text. The following sentence was added:

“To provide an overview of demographic and clinical characteristics of the study populations, Table 1 summarizes the participant characteristics across the included studies, while detailed study-level information is available in Supplementary Table S2.”

**2 ^nd^ Review: This is sufficiently addressed**

*Comment:* 3.3: While this is not a meta-analysis (and as such, there is not a need for formal analysis), it would strengthen the results to have a summary of findings in the studies (i.e. did pain scores and sleep scores correlate)? In addition, for pain outcomes, you note the predominance of NRS/VAS and BPI. Consider adding a summary matrix (main text or supplement) mapping pain domains (intensity, interference/disability, psychosocial, sensitization, QST) to the instruments encountered, indicating frequencies (some of this is described narratively on page 6)

*Response*:

 Thank you for this constructive suggestion. We agree that providing a summary of the associations and a structured mapping of assessment tools significantly strengthens the manuscript.

 Regarding the summary of findings, we have added a new section in the Results (3.4 Association between sleep and pain outcomes) to narratively describe the trends identified in the included studies. We reported that while patient-reported outcomes consistently demonstrated positive correlations between sleep and pain, objective measures showed distinct patterns: movement-based assessments (e.g., actigraphy) often lacked clear associations, whereas physiological measures (e.g., EEG) tended to correlate with pain outcomes. We have also added an interpretation of this discrepance to the Discussion section. The following sentences was added:

“3.4 Association between sleep and pain outcomes

Although a meta-analysis was not conducted, a narrative summary of the included studies reveals distinct patterns in the relationship between sleep and pain.

Studies relying on patient-reported outcomes consistently demonstrated a significant positive correlation, where higher score on sleep questionnaires (e.g., PSQI, ISI) were associated with greater pain intensity or interference.

In contrast, findings from objective assessments varied depending on the measurement modality. Studies utilizing movement-based devises, such as actigraphy, generally did not show a clear association with pain intensity or other pain-related outcomes. However, objective measures based on physiological parameters, such as heart rate or electroencephalography (EEG), tended to exhibit significant associations with pain outcomes.”

“Furthermore, our review identified a discrepancy in the results derived from different objective modalities. While movement-based assessment (e.g., actigraphy) often failed to correlate with pain, physiological measures (e.g., EEG, heart rate) consistently showed significant associations. This suggests that while pain may not always disrupt gross body movements during sleep, it likely impacts physiological sleep architecture and autonomic regulation.”

 Regarding the summary matrix, we have created a new table (Table 2) that maps the identified assessment tools to specific pain domains: Pain Intensity, Interference/Disability, Pain Quality, Psychosocial Factors, and Quantitative Sensory Testing. As suggested, this table presents the frequencies of use for each instrument within these domains. Please note that the Brief Pain Inventory (BPI) was categorized into both “Pain Intensity” and “Interference” domains, as it assesses both severity and impact on daily function.

**2 ^nd^ Review: The addition of section 3.4 adds important context to the manuscript. Our concerns are sufficiently addressed here.**

**Discussion**

*Comment*: The authors do well to highlight the heterogeneity in approach and the need for more standardized study design combining objective and subjective measures. However, I would like to see more discussion of what we do know about the relationship between sleep and pain [Reference 1], the potential impact of sleep deprivation on pain scores (PMID: 36334461) and hypothesize on mechanisms that may link the disorders [Reference 2].

 References

 1. Haack M, Simpson N, Sethna N, Kaur S, et al.: Sleep deficiency and chronic pain: potential underlying mechanisms and clinical implications. *Neuropsychopharmacology*. 2020; **45** (1): 205-216 Publisher Full Text

 2. Chang J, Fu S, Li X, Li S, et al.: The differential effects of sleep deprivation on pain perception in individuals with or without chronic pain: A systematic review and meta-analysis. *Sleep Medicine Reviews*. 2022; **66**. Publisher Full Text

*Response*:

 Thank you for your valuable feedback. We agree that elaborating on the established relationship and underlying mechanisms provides crucial context for our findings. We have revised the Discussion section to incorporate the suggested references. Specifically, we have expanded the paragraph discussing the sleep-pain interaction to explicitly address: 

1. The bidirectional nature of the relationship (Finan et al., 2013)
2. Experimental evidence showing that sleep deprivation significantly increases pain sensitivity (Chang et al., 2022)
3. Hypothesized neurobiological mechanisms, such as central sensitization and impaired descending pain inhibition (Haack et al., 2020; Nijs et al., 2018)

In accordance with these revisions, we have updated the reference list. The following sentences and references were added:

“Current evidence emphasizes a strong bidirectional relationship between sleep and pain, with sleep disturbances often predicting future pain severity more robustly than pain predicts sleep problems. ^6^ Experimental studies have further demonstrated that sleep deprivation directly exacerbates pain perception, lowering pain thresholds and increasing hyperalgesia ^121^ (new ref). These interactions are hypothesized to be mediated by shared neurobiological mechanisms, particularly central sensitization and impairments in descending pain inhibitory pathways, which nociceptive signaling. ^122,123^ Incorporating objective sleep assessments and multidimensional pain measures into research and practice will enhance the quality of evidence and support the development of precision medicine approaches tailored to individual patient needs.”

“121 Chang JR, Fu SN, Li X, et al. The differential effects of sleep deprivation on pain perception in individuals with or without chronic pain: A systematic review and meta-analysis. *Sleep Med Rev*. 2022;66:101695. 36334461 10.1016/j.smrv.2022.101695”

“123 Nijs J, Mairesse O, Neu D, et al.: Sleep Disturbances in Chronic Pain: Neurobiology, Assessment, and Treatment in Physical Therapist Practice. *Phys. Ther.*2018;98:325–335. 29425327 10.1093/ptj/pzy020”

**2 ^nd^ Review: We appreciate the addition of a mechanistic discussion of the b-directional. While it would be possible to have more depth, in the context of this manuscript, the above description sufficiently describes the underlying pathophysiology.**

 These revisions improve the clarity, methodological transparency, and interpretability of our review.

**Overall 2 ^nd^ Review: The authors have sufficiently addressed our concerns regarding the methodology, description of the results, and the depth of the discussion. We believe the manuscript is now suitable for Indexing.**

Are the rationale for, and objectives of, the Systematic Review clearly stated?

Yes

Is the statistical analysis and its interpretation appropriate?

Yes

If this is a Living Systematic Review, is the ‘living’ method appropriate and is the search schedule clearly defined and justified? (‘Living Systematic Review’ or a variation of this term should be included in the title.)

Not applicable

Are sufficient details of the methods and analysis provided to allow replication by others?

Yes

Are the conclusions drawn adequately supported by the results presented in the review?

Partly

Reviewer Expertise:

Sleep medicine, chronic pain, anesthesiology

We confirm that we have read this submission and believe that we have an appropriate level of expertise to confirm that it is of an acceptable scientific standard.

# Reviewer report: Reviewer response for version 3

**DOI:** 10.5256/f1000research.192814.r414411

**Published:** 2026-01-02

**Authors:** Brett D Neilson

**Affiliations:** 1 Hawai'i Pacific University, Hawaii, USA

Thank you to the authors for addressing my prior concerns and recommendations. No further feedback is required at this stage.

Are the rationale for, and objectives of, the Systematic Review clearly stated?

Yes

Is the statistical analysis and its interpretation appropriate?

Yes

If this is a Living Systematic Review, is the ‘living’ method appropriate and is the search schedule clearly defined and justified? (‘Living Systematic Review’ or a variation of this term should be included in the title.)

Not applicable

Are sufficient details of the methods and analysis provided to allow replication by others?

Yes

Are the conclusions drawn adequately supported by the results presented in the review?

Yes

Reviewer Expertise:

Physical therapy researcher whose scholarly agenda is focused on the intersection of sleep and pain. I believe I am well qualified to review and approve this manuscript.

I confirm that I have read this submission and believe that I have an appropriate level of expertise to confirm that it is of an acceptable scientific standard.

# Reviewer report: Reviewer response for version 2

**DOI:** 10.5256/f1000research.188083.r421813

**Published:** 2025-11-06

**Authors:** Yuri Chaves Martins, Peyton Murin

**Affiliations:** 1 Anesthesiology, Saint Louis University School of Medicine (Ringgold ID: 12274), St. Louis, Missouri, USA; 2 Neurology, Saint Louis University School of Medicine (Ringgold ID: 12274), St. Louis, Missouri, USA

The authors provide a systematic review looking at the relationship between sleep and chronic pain. The study follows JBI guidance, is OSF-registered, adheres to PRISMA-ScR, and synthesizes 81 studies from a multi-database search (finalized March 29, 2024). They use a robust AI assisted approach to identify articles meeting all screening, inclusion, and exclusion criteria. They find significant heterogeneity in measures for both pain and sleep disorders, highlighting a need for more consistency in study design/approach, a well-documented problem within chronic pain.

 Introduction:

 The authors summarize the background transitioning from chronic pain to sleep disorders. Identify the high prevalence of sleep disorders and chronic pain. Address the relationship between sleep and other comorbidities including pain and identify a gap in the literature.

 Minor: The transition between pain to chronic pain could be smoother. Would recommend rewording to allow for better flow/cohesion.

 Methods:

 Approach is appropriate. Inclusion and exclusion criteria are appropriate. Use of AI is appropriate and documented.

 You transparently describe using ChatGPT-4o and NotebookLM with human verification. To enhance reproducibility, please (i) provide the exact extraction template (e.g., the Excel column headings) in the OSF repository and (ii) add one or two concrete examples of verification checks you performed (e.g., double-checking instrument counts against full texts).

 The search was last run on March 29, 2024. You acknowledge that newer studies may be missing (page 7). Given the rapid growth in wearable-based sleep assessments, a short sensitivity statement (e.g., how many additional records a quick 2024–2025 update might add based on pilot scoping) would contextualize recency without requiring a full rerun.

 Results:

 3.1 : No concerns

 3.2: Would recommend including more robust analysis on the gender distribution, age, pain type. Would recommend adding a table to summarize this data.

 3.3: While this is not a meta-analysis (and as such, there is not a need for formal analysis), it would strengthen the results to have a summary of findings in the studies (i.e. did pain scores and sleep scores correlate)? In addition, for pain outcomes, you note the predominance of NRS/VAS and BPI. Consider adding a summary matrix (main text or supplement) mapping pain domains (intensity, interference/disability, psychosocial, sensitization, QST) to the instruments encountered, indicating frequencies (some of this is described narratively on page 6)

 Discussion:

 The authors do well to highlight the heterogeneity in approach and the need for more standardized study design combining objective and subjective measures. However, I would like to see more discussion of what we do know about the relationship between sleep and pain [Reference 1], the potential impact of sleep deprivation on pain scores (PMID: 36334461) and hypothesize on mechanisms that may link the disorders [Reference 2].

Are the rationale for, and objectives of, the Systematic Review clearly stated?

Yes

Is the statistical analysis and its interpretation appropriate?

Yes

If this is a Living Systematic Review, is the ‘living’ method appropriate and is the search schedule clearly defined and justified? (‘Living Systematic Review’ or a variation of this term should be included in the title.)

Not applicable

Are sufficient details of the methods and analysis provided to allow replication by others?

Yes

Are the conclusions drawn adequately supported by the results presented in the review?

Partly

Reviewer Expertise:

Sleep medicine, chronic pain, anesthesiology

We confirm that we have read this submission and believe that we have an appropriate level of expertise to confirm that it is of an acceptable scientific standard, however we have significant reservations, as outlined above.

## References

[1] : Sleep deficiency and chronic pain: potential underlying mechanisms and clinical implications. *Neuropsychopharmacology* .2020;45(1) : 10.1038/s41386-019-0439-z 205-216 10.1038/s41386-019-0439-z PMC687949731207606

[2] : The differential effects of sleep deprivation on pain perception in individuals with or without chronic pain: A systematic review and meta-analysis. *Sleep Medicine Reviews* .2022;66: 10.1016/j.smrv.2022.101695 10.1016/j.smrv.2022.101695 36334461

# Response

**Published:** 2025-12-02

**Authors:** Katsuyoshi Tanaka

**Response to Yuri Chaves Martins**

 We greatly appreciate the reviewer’s insightful comments. Our responses to your comments are given below.

**Introduction**

*Comment:* The transition between pain to chronic pain could be smoother. Would recommend rewording to allow for better flow/cohesion.

*Responses:*

 We appreciate the reviewer’s helpful suggestion. To improve the logical flow from pain to chronic pain, we have revised the beginning of the Introduction. Specifically, we added a sentence to clarify the conceptual transition from pain as a protective mechanism to chronic pain as a pathological condition. The following text was added:

“Pain is a fundamental human experience that functions as a protective mechanism, however, when it persists beyond the expected period of tissue healing, it is regarded as a pathological condition known as chronic pain.”

**Methods**

*Comment:* You transparently describe using ChatGPT-4o and NotebookLM with human verification. To enhance reproducibility, please (i) provide the exact extraction template (e.g., the Excel column headings) in the OSF repository and (ii) add one or two concrete examples of verification checks you performed (e.g., double-checking instrument counts against full texts).

 The search was last run on March 29, 2024. You acknowledge that newer studies may be missing (page 7). Given the rapid growth in wearable-based sleep assessments, a short sensitivity statement (e.g., how many additional records a quick 2024–2025 update might add based on pilot scoping) would contextualize recency without requiring a full rerun.

*Responses:*

 We sincerely thank the reviewer for these valuable and constructive comments.

 (i) To improve transparence and reproducibility, we have updated the Excel data extraction template (showing the column headings used for data collection) to our Open Science Framework (OSF) repository ( https://doi.org/10.17605/OSF.IO/5JK63). The revised Methods section has showed the major data fields extracted (eg., author country, study design, and tools used for sleep and pain assessment). The following sentence was added:

“To enhance reproducibility, the exact data extraction template has been made publicly available in the Open Science Framework repository ( https://doi.org/10.17605/OSF.IO/5JK63 ). The column headings include, for example, “Title”, “Author”, “Year of publication”, “Country”, “Study design”, “Diagnosis/Conditions”, “Sample size”, “Age”, “Sleep assessment tool”, and “Assessment tools of pain-related factor”.”

 (ii) We have also added specific examples of verification procedures used during data validation. These included double-checking the counts of sleep and pain assessment tools extracted by AI against both the full-text tables and textual descriptions, and cross-referencing the extracted sample characteristics with the methods section. The following sentence was added:

“Verification checks included double-checking the counts of sleep and pain assessment tools extracted by AI against both the full-text tables and textual descriptions, and cross-referencing the extracted sample characteristics with the methods sections of the original papers. ”

 (iii) To contextualize the recency of our research, we performed an updated PubMed search using the same search strategy as in the original review, limited to publications from 2024 to 2025, on November 7, 2025. This updated search identified 131 new records, reflecting a moderate increase in studies related to wearable-based sleep assessments. We have added a corresponding sensitivity statement to the Methods section, clarifying that the findings of the present review remain broadly representative of the current evidence base, while future updates should incorporate these recent studies. The following sentences were added:

“The literature search was last conducted on March 29, 2024. To contextualize the recency of the review, an updated search was performed in PubMed using the same search strategy, limited to publications from 2024 to 2025, on November 7, 2025. This additional search identified 131 new records. Although this increase indicates an ongoing rise in research activity, particularly in wearable-based sleep assessments, the number of new studies remains modest relative to the total volume of literature included in the present review. Therefore, the findings of this review are considered broadly representative of the current evidence base, while future updates should incorporate these recent studies to capture emerging trends. ”

**Results**

*Comment:* 3.2: Would recommend including more robust analysis on the gender distribution, age, pain type. Would recommend adding a table to summarize this data.

*Response*:

 Thank you for this helpful suggestion. In response, we strengthened the description of participant characteristics by providing an aggregated summary of age distribution, sex distribution, and pain diagnoses in the main text. The following sentence was added:

“To provide an overview of demographic and clinical characteristics of the study populations, Table 1 summarizes the participant characteristics across the included studies, while detailed study-level information is available in Supplementary Table S2.”

*Comment:* 3.3: While this is not a meta-analysis (and as such, there is not a need for formal analysis), it would strengthen the results to have a summary of findings in the studies (i.e. did pain scores and sleep scores correlate)? In addition, for pain outcomes, you note the predominance of NRS/VAS and BPI. Consider adding a summary matrix (main text or supplement) mapping pain domains (intensity, interference/disability, psychosocial, sensitization, QST) to the instruments encountered, indicating frequencies (some of this is described narratively on page 6)

*Response*:

 Thank you for this constructive suggestion. We agree that providing a summary of the associations and a structured mapping of assessment tools significantly strengthens the manuscript.

 Regarding the summary of findings, we have added a new section in the Results (3.4 Association between sleep and pain outcomes) to narratively describe the trends identified in the included studies. We reported that while patient-reported outcomes consistently demonstrated positive correlations between sleep and pain, objective measures showed distinct patterns: movement-based assessments (e.g., actigraphy) often lacked clear associations, whereas physiological measures (e.g., EEG) tended to correlate with pain outcomes. We have also added an interpretation of this discrepance to the Discussion section. The following sentences was added:

“3.4 Association between sleep and pain outcomes

Although a meta-analysis was not conducted, a narrative summary of the included studies reveals distinct patterns in the relationship between sleep and pain.

Studies relying on patient-reported outcomes consistently demonstrated a significant positive correlation, where higher score on sleep questionnaires (e.g., PSQI, ISI) were associated with greater pain intensity or interference.

In contrast, findings from objective assessments varied depending on the measurement modality. Studies utilizing movement-based devises, such as actigraphy, generally did not show a clear association with pain intensity or other pain-related outcomes. However, objective measures based on physiological parameters, such as heart rate or electroencephalography (EEG), tended to exhibit significant associations with pain outcomes.”

“Furthermore, our review identified a discrepancy in the results derived from different objective modalities. While movement-based assessment (e.g., actigraphy) often failed to correlate with pain, physiological measures (e.g., EEG, heart rate) consistently showed significant associations. This suggests that while pain may not always disrupt gross body movements during sleep, it likely impacts physiological sleep architecture and autonomic regulation.”

 Regarding the summary matrix, we have created a new table (Table 2) that maps the identified assessment tools to specific pain domains: Pain Intensity, Interference/Disability, Pain Quality, Psychosocial Factors, and Quantitative Sensory Testing. As suggested, this table presents the frequencies of use for each instrument within these domains. Please note that the Brief Pain Inventory (BPI) was categorized into both “Pain Intensity” and “Interference” domains, as it assesses both severity and impact on daily function.

**Discussion**

*Comment*: The authors do well to highlight the heterogeneity in approach and the need for more standardized study design combining objective and subjective measures. However, I would like to see more discussion of what we do know about the relationship between sleep and pain [Reference 1], the potential impact of sleep deprivation on pain scores (PMID: 36334461) and hypothesize on mechanisms that may link the disorders [Reference 2].

 References

 1. Haack M, Simpson N, Sethna N, Kaur S, et al.: Sleep deficiency and chronic pain: potential underlying mechanisms and clinical implications. *Neuropsychopharmacology*. 2020; **45** (1): 205-216 Publisher Full Text

 2. Chang J, Fu S, Li X, Li S, et al.: The differential effects of sleep deprivation on pain perception in individuals with or without chronic pain: A systematic review and meta-analysis. *Sleep Medicine Reviews*. 2022; **66**. Publisher Full Text

*Response*:

 Thank you for your valuable feedback. We agree that elaborating on the established relationship and underlying mechanisms provides crucial context for our findings. We have revised the Discussion section to incorporate the suggested references. Specifically, we have expanded the paragraph discussing the sleep-pain interaction to explicitly address:

1. The bidirectional nature of the relationship (Finan et al., 2013)
2. Experimental evidence showing that sleep deprivation significantly increases pain sensitivity (Chang et al., 2022)
3. Hypothesized neurobiological mechanisms, such as central sensitization and impaired descending pain inhibition (Haack et al., 2020; Nijs et al., 2018)

In accordance with these revisions, we have updated the reference list. The following sentences and references were added:

“Current evidence emphasizes a strong bidirectional relationship between sleep and pain, with sleep disturbances often predicting future pain severity more robustly than pain predicts sleep problems. ^6^ Experimental studies have further demonstrated that sleep deprivation directly exacerbates pain perception, lowering pain thresholds and increasing hyperalgesia ^121^ (new ref). These interactions are hypothesized to be mediated by shared neurobiological mechanisms, particularly central sensitization and impairments in descending pain inhibitory pathways, which nociceptive signaling. ^122,123^ Incorporating objective sleep assessments and multidimensional pain measures into research and practice will enhance the quality of evidence and support the development of precision medicine approaches tailored to individual patient needs.”

“121 Chang JR, Fu SN, Li X, et al. The differential effects of sleep deprivation on pain perception in individuals with or without chronic pain: A systematic review and meta-analysis. *Sleep Med Rev*. 2022;66:101695. 36334461 10.1016/j.smrv.2022.101695”

“123 Nijs J, Mairesse O, Neu D, et al.: Sleep Disturbances in Chronic Pain: Neurobiology, Assessment, and Treatment in Physical Therapist Practice. *Phys. Ther.* 2018;98:325–335. 29425327 10.1093/ptj/pzy020”

 These revisions improve the clarity, methodological transparency, and interpretability of our review.

# Reviewer report: Reviewer response for version 1

**DOI:** 10.5256/f1000research.182981.r399708

**Published:** 2025-08-27

**Authors:** Brett D Neilson

**Affiliations:** 1 Hawai'i Pacific University, Hawaii, USA

General Comments

 This is a well-organized and clearly written scoping review (ScR) that addresses an important topic: the methods used to assess sleep and pain-related outcomes in individuals with chronic pain. The authors articulate a clear aim, follow a structured methodology, and present their findings with clarity. The manuscript would benefit from some clarifications and adjustments, particularly regarding terminology, methodological rigor, and reporting of results.

 Abstract

- The abstract is clear and concisely conveys the purpose and scope of the review.
- The aim is well articulated.
- No major revisions are needed for the abstract.

Introduction

 The introduction is generally well written and provides appropriate context for the review. However, the use of inconsistent terminology for sleep-related issues may create confusion for the reader.

 Recommendations:

- Consider defining or consistently using terms such as “sleep problems,” “sleep disorders,” “compromised sleep,” and “sleep disturbances.”
- Reference standardized definitions or diagnostic criteria where appropriate.
- The statement: “The financial impact of chronic pain, including healthcare expenses and reduced work efficiency, is substantial.” should be supported with a citation. There is robust literature available on this topic.

Methods

 The methodology appears rigorous, with an extensive search strategy across nine databases and inclusion of grey literature. However, a few clarifications and enhancements are suggested.

 Recommendations:

- Clarify adherence to all eight JBI steps, or note deviations.
- Consider referencing the PRISMA-ScR checklist.
- Search was completed in March 2024; consider updating if required by the journal.
- Describe how ChatGPT-4o outputs were validated to ensure data accuracy.
- Mention if data extraction software (e.g., Covidence) was used.
- Include a sample search strategy for at least one database to enhance reproducibility of the review process.

Results

 The results are clearly reported, with appropriate use of tables and figures.

 Recommendation:

- Provide percentages or refer to Figure 2 when discussing the use of PSQI and ISI.

Discussion

 The discussion section is well constructed and effectively summarizes the key findings of the review.

 Recommendations:

- Use consistent and defined terminology for sleep-related terms.
- Discuss how inadequate sleep assessment may affect intervention outcomes, given the bidirectional relationship between sleep and pain.

Conclusions

 The conclusions are appropriate and do not overreach.

 Recommendation:

- Replace the term “content” with “characteristics” or “types” where appropriate.

Summary of Key Revisions for Authors

- Ensure consistent and defined terminology for sleep-related dysfunction.
- Add citation(s) to support claims regarding the financial impact of chronic pain.
- Clarify adherence to JBI methodology and consider referencing PRISMA-ScR.
- Provide detail on how ChatGPT-4o was used and how its outputs were validated.
- Include numerical data or clear reference to figures for key result statements.
- Explore how measurement inconsistencies could impact intervention outcomes.
- Replace the term “content” with “characteristics” or “types” where appropriate.
- Acknowledge that the PRISMA-ScR checklist was used, as mentioned in the Data Availability section. Consider integrating this earlier in the Methods to emphasize adherence to reporting standards.
- Include a sample search strategy for at least one database to enhance reproducibility of the review process.

Are the rationale for, and objectives of, the Systematic Review clearly stated?

Yes

Is the statistical analysis and its interpretation appropriate?

Yes

If this is a Living Systematic Review, is the ‘living’ method appropriate and is the search schedule clearly defined and justified? (‘Living Systematic Review’ or a variation of this term should be included in the title.)

Not applicable

Are sufficient details of the methods and analysis provided to allow replication by others?

Yes

Are the conclusions drawn adequately supported by the results presented in the review?

Yes

Reviewer Expertise:

I am a physical therapist educator in the Hawai‘i Pacific University Doctor of Physical Therapy Program. My research focuses on the intersection of sleep and pain, with several publications in this area. I have previously published two ScRs on a similar topic: Feda J, Miller T, Young JL, Neilson B, Rhon DI. Measures of sleep are not routinely captured in trials assessing treatment outcomes in knee osteoarthritis - A scoping systematic review and call to action. Osteoarthr Cartil Open. 2023;(100400):100400. Neilson BD, Dickerson C, Young JL, Shepherd MH, Rhon DI. Measures of sleep disturbance are not routinely captured in trials for chronic low back pain: a systematic scoping review of 282 trials. J Clin Sleep Med. 2023;19(11):1961–1970.

I confirm that I have read this submission and believe that I have an appropriate level of expertise to confirm that it is of an acceptable scientific standard, however I have significant reservations, as outlined above.

# Response

**Published:** 2025-09-12

**Authors:** Katsuyoshi Tanaka

**Response to Brett D Neilson**

 We greatly appreciate the reviewer’s insightful comments. Our responses to your comments are given below.

**Introduction**

*Comment:* The introduction is generally well written and provides appropriate context for the review. However, the use of inconsistent terminology for sleep-related issues may create confusion for the reader.

*Recommendations:*

- Consider defining or consistently using terms such as “sleep problems,” “sleep disorders,” “compromised sleep,” and “sleep disturbances.”
- Reference standardized definitions or diagnostic criteria where appropriate.
- The statement: “The financial impact of chronic pain, including healthcare expenses and reduced work efficiency, is substantial.” should be supported with a citation. There is robust literature available on this topic.

*Responses:*

 We appreciate the reviewer’s suggestion. To avoid confusion, we have revised the manuscript to use the term “sleep disturbances” consistently when referring to general sleep-related dysfunction. In the Introduction, we also added a clarifying statement with references to the International Classification of Sleep Disorders, Third Edition (ICSD-3).

 Additionally, as recommended, we have added a citation to support the statement regarding the financial impact of chronic pain.

 We have added the references as follow:

“ Rikard SM, Strahan AE, Schmit KM, Guy GP Jr. Chronic pain among adults - United States, 2019-2021. *MMWR Morb Mortal Wkly Rep*. 2023;72:379-385. ”

“ Sateia MJ. International classification of sleep disorders-third edition: highlights and modifications. *Chest*. 2014;146:1387-1394. ”

**Methods**

*Comment:* The methodology appears rigorous, with an extensive search strategy across nine databases and inclusion of grey literature. However, a few clarifications and enhancements are suggested.

*Recommendations:*

1. Clarify adherence to all eight JBI steps, or note deviations.
2. Consider referencing the PRISMA-ScR checklist.
3. Search was completed in March 2024; consider updating if required by the journal.
4. Describe how ChatGPT-4o outputs were validated to ensure data accuracy.
5. Mention if data extraction software (e.g., Covidence) was used.
6. Include a sample search strategy for at least one database to enhance reproducibility of the review process.

*Responses:*

 We appreciate the reviewer’s suggestions. We have responded to each recommendation as below:

 1. Adherence to JBI steps

 We clarified that our review followed all eight steps of the Joanna Briggs Institute (JBI) methodology for scoping review without deviation. We revised the sentence as below:

“This ScR was conducted according to the Joanna Briggs Institute methodology for scoping reviews, following all eight recommended steps without deviation.”

 2. PRISMA-ScR checklist

 We appreciate the reviewer’s recommendation. In addition to noting in the Data Availability section that the completed PRISMA-ScR checklist is provided, we have now explicitly stated in the Methods section that the review adhered to the PRISMA-ScR checklist. The following sentence was added:

“This review also adhered to the Preferred Reporting Items for Systematic Reviews and Meta-Analyses extension for Scoping reviews (PRISMA-ScR) checklist.”

 3. Search update

 We appreciate the reviewer’s suggestion. The final comprehensive search was conducted on March 29, 2024, as already noted in the Methods section. Given the publication model of F1000Research, the Version 1 preprint of this review was made publicly available shortly after submission, and thus the search reflects the literature available at that time. As the journal does not require an updated search for scoping reviews, we have not repeated the search. Nevertheless, we have noted in the Discussion that more recent studies may not have been captured. The following sentence was added:

“A limitation of this review is that the literature search was last conducted in March 2024, and more recent studies may not have been capture.”

 4. Use of ChatGPT-4o

 We thank the reviewer for this important comment. In the revised manuscript, we have clarified how ChatGPT-4o and NotebookLM were used during data extraction. These AI tools were used to facilitate the extraction and preliminary organization of information from the included studies. All outputs were reviewed and verified against the original studies by the first author (K.T.) to ensure accuracy. Thus, while AI was utilized as part of the data extraction process, no information was included without human verification, and all final decisions were made by the research team. The following sentence was added:

“These AI tools were used to facilitate the extraction and preliminary organization of information from the included studies. All outputs generated by AI were reviewed and verified against the original sources by the first author (K.T.) to ensure accuracy, and all final decisions were made by the research team.”

 5. Data extraction software

 We thank the reviewer’s helpful comment. In the revised manuscript, we have clarified that no dedicated systematic review software such as Covidence was used for data extraction. Instead, data were extracted using Microsoft Excel, supported by ChatGPT-4o and NotebookLM, with all outputs reviewed and verified by the first author (K.T.) to ensure accuracy. The following sentence was added:

“No dedicated systematic review software (e.g., Covidence) was used for data extraction.”

 6. Sample search strategy

 We thank the reviewer’s valuable suggestion. To ensure reproducibility, we have clarified in the Methods section that the full PubMed search strategy is provided as Supplementary Table S1. The sentence was revised as follow:

“The text words found in the titles and abstracts of relevant articles, along with the index terms used to describe the articles, were used to develop a comprehensive search strategy across nine databases (the complete PubMed search strategy is provided in Table S1).”

**Results**

*Comment: *The results are clearly reported, with appropriate use of tables and figures.

*Recommendation:*

- Provide percentages or refer to Figure 2 when discussing the use of PSQI and ISI.

Response:

 We appreciate the reviewer’s suggestion. To improve clarity, we have added the frequencies and percentages of studies that used the PSQI and ISI, and we now reference Figure 2 in the text. We revised the sentences as follow:

 “ The Pittsburgh Sleep Quality Index (PSQI) (45 of 81 studies, 55.6%) and Insomnia Severity Index (ISI) (21 of 81 studies, 25.9%) were the most commonly used tools for the assessment of sleep problems (Figure 2).”

**Discussion**

*Comment:* The discussion section is well constructed and effectively summarizes the key findings of the review.

*Recommendations:*

- Use consistent and defined terminology for sleep-related terms.
- Discuss how inadequate sleep assessment may affect intervention outcomes, given the bidirectional relationship between sleep and pain.

*Responses:*

 We appreciate the reviewer’s comments. In the revised manuscript, we have ensured consistent use of the term “sleep disturbances” in the Discussion section. We have also added text to highlight how inadequate or inconsistent sleep assessment may influence the interpretation of intervention outcomes, given the bidirectional relationship between sleep and pain. The following sentences were added:

“Given the bidirectional relationship between sleep and pain, inadequate or inconsistent assessment of sleep disturbances may obscure the true effects of intervention for chronic pain. This limitation could lead to underestimation or misinterpretation of treatment efficacy and reduce the comparability of results across studies. Addressing this issue is essential for developing effective multimodal interventions and advancing precision medicine.”

**Conclusions**

*Comment:* The conclusions are appropriate and do not overreach.

*Recommendation:*

- Replace the term “content” with “characteristics” or “types” where appropriate.

*Response:*

 We appreciate the reviewer’s suggestion. In the revised manuscript, we have replaced the term content with characteristics in the Conclusions section to improve clarity as follows:

“This ScR highlights the imbalance in the characteristics of sleep and pain assessments, indicating the need for a more comprehensive evaluation of sleep disturbances and pain-related outcomes.”

**Summary of Key Revisions**

 We sincerely thank the reviewer for the thorough and constructive feedback. In response to the comments, we have made the following key revisions to the manuscript:

1. **Terminology**: We ensured consistent use of the term sleep disturbances across the Introduction and Discussion, and provided standardized definitions with citations to the ICSD-3.
2. **Introduction**: We added a citation to support the statement regarding the financial impact of chronic pain.
3. **Methods**:
  - Clarified adherence to all eight JBI steps.
  - Explicitly stated adherence to the PRISMA-ScR checklist.
  - Explained that the final search was conducted in March 2024 and noted this as a limitation.
  - Clarified how ChatGPT-4o and NotebookLM were used to facilitate data extraction, and described human verification procedures.
  - Stated that no dedicated systematic review software (e.g., Covidence) was used.
  - Specified that the complete PubMed search strategy is provided in Supplementary Table S1.
4. **Results**: Added the frequencies and percentages of studies using the PSQI and ISI, with reference to Figure 2.
5. **Discussion**: Ensured consistent terminology and added a statement on how inadequate sleep assessment may affect the interpretation of intervention outcomes, given the bidirectional relationship between sleep and pain.
6. **Conclusions**: Replaced the term content with characteristics for improved clarity.

These revisions improve the clarity, methodological transparency, and interpretability of our review.
